# Teacher support in language learning: a picture of the effects on language progress, academic immunity, and academic enjoyment

**DOI:** 10.1186/s40359-024-01602-2

**Published:** 2024-03-04

**Authors:** Lan Huang, Anwar Hammad Al-Rashidi, Sania Bayat

**Affiliations:** 1 School of Humanities and Foreign Languages, Hunan Agricultural University, 410125 Hunan, China; 2 Department of Psychology, College of Education, Prince Sattam Bin Abdulaziz University, Al-Kharj, Saudi Arabia; 3 Department of English Literature, Faculty of Foreign Languages, Rasht Branch, Islamic Azad University, Rasht, Iran

**Keywords:** Teacher support, Language progress, Academic immunity, Academic enjoyment, EFL learners

## Abstract

Teacher support encompasses the provision of educational, instrumental, sentimental, or evaluative assistance by a teacher to a student, regardless of the setting. Learning a new language brings with it different ups and downs that may hinder the actual progress of the learners. The journey of learning may be made easier by supportive instructors. During the course of this inquiry, two groups (85 participants in total) including the experimental and control groups were chosen to gauge the influence of Teacher support in virtual instruction on English as a foreign language (EFL) learners’ progress, academic immunity, and academic enjoyment. In contrast to the experimental group (EG, *n* = 42), which had the chance to connect with a teacher in order to ask questions and get feedback plus the off-line education, the control group (CG, *n* = 44) was subjected to off-line instruction of language input (Top Notch 3). The status of the participants’ language growth, immunity, and enjoyment was investigated both before and after the treatment. The findings of independent samples t-tests as well as the multivariate analysis of variance (MANOVA) indicated that learners in EG did much better than their counterparts in CG. In addition, the ramifications and recommendations for potential future directions are analyzed.

**Supplementary Information:**

The online version contains supplementary material available at 10.1186/s40359-024-01602-2.

## Introduction

In language classrooms, instructors are often considered a crucial component alongside students and course materials [1, 2]. The teacher-student connection is seen as a crucial mechanism that may enhance students’ engagement, motivation, and resilience via the provision of a supportive and positive learning environment [3, 4]. Teachers may create a safe and supportive classroom climate by getting to know their students and meeting their needs [5]. Support for teachers has been the subject of an increasing corpus of study in the fields of education and educational psychology, which has verified the significant advantages that it provides for students. For instance, studies demonstrate that teacher support can substantially improve students’ learning outcomes, enthusiasm, mastering strategy use, innovative perception, perseverance, engagement, dedication to developing tasks, and professional advancement [6]. On top of that, a number of studies have shown that increased levels of support from teachers might motivate students to commit more time to their studies.

The existing body of literature regarding the influence of support from significant individuals, particularly EFL teachers, remains relatively limited and disjointed. This is despite the acknowledgment that language learners function as proactive and self-reflective agents engaged in interactions within their social context [7]. An important component of successful foreign language learning is students’ desire to speak, and research has shown that teachers’ encouragement may greatly increase this willingness [1, 8]. Furthermore, research has shown that when teachers are there to support their students, it can have a positive impact on their motivation, engagement, learning experience, emotions, and overall well-being. This includes boosting positive emotions like enthusiasm and optimism, as well as effective learning, and alleviating negative emotions like anxiety by creating a safe space in class. Also, among EFL students, [7] found that students’ perceptions of their teachers’ support were positively correlated with both their academic performance and their participation in group projects. Moreover, with enough encouragement from EFL instructors, students may strengthen their ability to bounce back from adversity and conquer the difficult tasks that come with learning a new language [8]. Ultimately, students’ interest and effort levels are strong predictors of their foreign language achievement [9], which is especially important given the demanding and challenging nature of learning a foreign language over a long period of time [7, 8].

Academic enjoyment (AE) is an important attribute in education that influences learners’ pleasure in language acquisition [9]. AE encourages students to respond enthusiastically and builds emotional resilience [10]. AE is intimately connected to and a forerunner to cultivating a feeling of performance [11, 12]. However, creating a favorable learning environment and using effective tactics are required for effective language acquisition. That is, to create a successful learning environment, instructors should create a climate in which AE is developed and class activities and evaluations contribute to learners’ sentiments of satisfaction.

Another key concept is AI, which vaccinated learners against educational issues and difficulties [13]. AI is a psychological and sociological concept [14] and functions as a defensive mechanism against the high-intensity chaos and complexity of educational contexts. It has an impact on pupils’ ideas, emotions, and actions from a psychological standpoint. It formulates how students interact and behave in the learning environment from a sociological standpoint [15]. To be more specific, productive immunity plays a crucial role in individuals’ well-being by acting as a protective barrier against stress, failure, burnout, anxiety, and other adverse influences [16]. Despite the considerable potential for extensive research in this domain, as far as the researcher is aware, no study has been conducted to date to uncover the connections between TS in virtual instruction, language advancement, AE, and AI, particularly in the context of EFL environments. The findings of this research may open up new avenues for improved understanding of TS, language progress, AE, and AI, as well as their roles in language education.

## Literature review

Immunity is a nascent notion in the field of education. AI, like biological immunity, may have both productive and mal-productive effects [13]. Productive immunity serves as a defense mechanism against negative emotions, such as anxiety, fear, and challenges. On the other hand, maladaptive immunity arises from learners’ inability to adapt to new ideas or changes, as well as the failure of self-regulatory strategies to function properly [14]. The concept of immunity within the educational system is strengthened by the self-organization theory, which is derived from the complexity theory [17]. The idea of self-organization posits that people have the capacity to modify their internal mechanisms in response to external obstacles in order to maintain their survival [18].

The research lacuna in the field of AI is almost identical, and its correlation has not been investigated. An investigation was made by [15] into the immunology of language learners [15]. aimed to investigate the immunology of individuals who are learning a new language. The claim is made that LI is an educational, social, and psychological framework that enables learners to take action against issues and challenges. Similarly, [19] found that higher levels of reflective thinking skills were associated with greater satisfaction and productive immunity among EFL students, according to their findings. Moreover, the results might open up new avenues for integrating thinking skills techniques and learning-oriented evaluation into educational initiatives.

AE, as defined in positive psychology, is the emotional response that arises from achieving a goal or completing a task successfully [20]. AE is a complex concept that encompasses five different processes: psychological, cognitive, inspirational, interpersonal, and corporeal [21]. AE has an impact on learners’ motivation, social interactions with peers and instructors, and physical health [22, 23]. The event of enjoyment is inherently a fluid construct. Regarding this matter, [24] confirmed that language AE changes throughout time based on the personality of the language learners as well as the educational setting in which they are learning the language. It has been shown in the research that the interactions between teachers and students have a significant role in the process of fostering AE in the classroom [24–26]. The equilibrium between context-oriented characteristics and the mental requirements of the students is another factor that contributes to the satisfaction of the classroom [27]. A similar line of reasoning may be seen in the findings of [28], which demonstrate that students who report high levels of pleasure are more likely to speak out and interact with their peers in the classroom. In a further development, [11] penned a review article in order to demonstrate the satisfaction and engagement associated with learning a foreign language. AE has been shown to boost learners’ academic motivation and engagement, which in turn ensures that learners will continue to achieve their goals over time.

## Theoretical framework

The social support paradigm is fundamental to teacher support [29]. proposed a five-part model for social support, which includes guidance (the act of providing or receiving support), description/evaluation (the act of describing or assessing social support), attitude (the state of being available or used), materials (support that is instrumental, educational, sentimental, or appraisal support), and an assistance network. Students may get social support from their instructors, classmates, and family [30]. Teachers may give several sorts of support, such as informational, emotional, instrumental, and evaluation assistance [31, 32]. Teachers’ giving of information, guidance, or counsel on a certain topic is referred to as informational assistance. Empathy, affection, belonging, and trust are all associated with emotional support. Time, money, and expertise are examples of instrumental assistance. Finally, assessment assistance refers to instructors providing evaluative comments and/or instruction to pupils in order to improve their performance.

While TS, AI, and AE have individually shown their effectiveness in helping students and thereby enhancing their educational achievement, no previous study has examined the connections among them. This research aimed to assess the role of TS in virtual education and how it improves EFL learners’ language achievement, AI, and AE. This study’s findings may be advantageous for both learners and educators in both theoretical and practical aspects. Based on these perspectives, the following queries are proposed in this investigation:

RQ1. Does TS provide insight into the language progress among EFL learners?

RQ2. Does TS provide insight into AI among EFL learners?

RQ3. Does TS provide insight into AE among EFL learners?

## Methodology

### Participants and procedures

A cohort of 85 participants, who participated in EFL instruction at a private language institute in Iran, were included in this research study. The selection of language learners was based on the outcomes of the Oxford Quick Placement Test. The age range of the learners was between 21 and 28 years old. According to their program of study, they were expected to successfully complete Top Notch 3 within 16 sessions. The participants in this study willingly and voluntarily provided their informed consent to participate in this inquiry.

### Measures

The student’s English proficiency was assessed using the Oxford Quick Placement Test (OQPT) (See Additional File 1). Students who achieved scores ranging from 0.7 to 0.9 on this exam, which has potential values ranging from 0.1 to 0.9, are considered to possess English language abilities at an upper intermediate level. The OQPT underwent a Cronbach’s alpha reliability test, with good results indicating a reliability of 0.91.

The participants took a test designed by the researcher based on the topics of Top Notch 3 both before and after the study to get insight into their language development. This test consisted of 48 questions, 12 in each of the areas of speaking, writing, listening, and reading. The test’s content and face validity were assessed by three psychometricians and two EFL instructors; the findings guided changes to the instrument. Then, using a sample of 33 EFL students at the upper intermediate level of English competency, the test-retest reliability was investigated. To make sure the findings persisted over time, the same test was administered to the same person again a few weeks later. The reported Pearson’s R (*r* = 0.913, *p* < 05) was extremely useful.

To inspect the participants’ immunity, the researchers in this study introduced modifications to the Language Teacher Immunity Instrument, adapting it to better suit the specific needs and objectives of their research, which was originally created and validated by [15]. The revised version, known as the Academic Immunity Instrument (AII), consists of seven subscales with a total of 39 questions. Participants rated the items on a 6-point Likert scale, ranging from 1 (strongly disagree) to 6 (strongly agree). The subscales within the Assessment of Individual Immunity (AII) included the following dimensions: learning self-efficacy (7 items), burnout (5 items), resilience (5 items), attitudes toward learning (5 items), openness to change (6 items), classroom affectivity (6 items), and coping (5 items). The reliability of the AII was evaluated, and the Cronbach alpha coefficient yielded a satisfactory value of α = 0.873, indicating a high level of internal consistency.

EFL learners’ enjoyment was assessed using the Foreign Language Enjoyment Scale (FLES), a tool developed and validated by [22]. The FLES employs a 5-point Likert scale with 21 items, where responses range from strongly disagree to strongly agree. In the context of this study, Cronbach’s alpha score for FLES was deemed appropriate (α = 0.873), indicating a satisfactory level of internal consistency.

### Data collection and analysis

The students’ English proficiency was first assessed using the OQPT. An intermediate level of English proficiency was defined as a score between 0.4 and 0.6. The researchers culled the class that scored poorly (between 0.1 and 0.4) and those that scored well (between 0.7 and 0.9) on the language competency scale. In the end, 85 students were divided into two groups: CG (*n* = 44) and EG (*n* = 42). The researchers used a quasi-experimental approach in their study. So that the findings might be trusted, the participants in this study were told not to take any extra English classes. A pre-test was conducted before administering the therapy. Five parts to this test measure the reflections of the participants on TS, language growth, AI, and AE.

The second step was to start practicing. In 2023, EG and CG met for 16 sessions, or one semester, to learn Top Notch 3. Off-line classes were held in CG and EG using a Telegram Channel, designed specifically for teaching the sections in Top Notch 3. The students in EG, in addition, had this opportunity to experience the teachers’ support via a private group in telegram. The teacher in this study provided EG with assistance and answered their queries throughout the course of the investigation. A diary in which EG might record their thoughts and reflections on their language acquisition was also suggested. It took sixteen sessions, or one semester, to complete this project. To evaluate the project’s efficacy and the EFL students’ development, CG and EG were administered a post-exam in session 16, just before the participants’ final evaluation. This test included Participants’ thoughts on TS, language growth, AI, and AE. Both pre- and post-tests were held online. Three EFL instructors checked the reliability of the outcomes by comparing the pre-and post-tests.

The effectiveness of TS on language growth, AI, and AE in off-line instruction was evaluated by Independent Samples t-test as well as one-way MANOVA. Before conducting MANOVA, several related assumptions, including normality, sample size, outliers, linearity, and homogeneity of regression, were thoroughly examined.

## Results

Three steps were followed for data analysis. The first step was checking the normality distribution of the data which was done through running the Kolmogorov-Smirnov test. It was revealed that the data are normal (*p* > 0.05) and using parametric statistics is safe in this research. Secondly, it was needed to ensure if any difference existed between the two groups before the treatment. To make certain about this, the researchers run an independent samples t-test as shown in Tables 1 and 2 below:

**Table 1:** Descriptive statistics (groups’ pretests of LP, AI, and AE)

	Groups	N	Mean	Std. Deviation	Std. Error Mean
LP Pretest	EG	42	29.4524	6.68527	1.03156
	CG	44	29.5682	8.34266	1.25770
AI Pretest	EG	42	122.6190	12.54252	1.93535
	CG	44	128.8409	23.38052	3.52475
AE Pretest	EG	42	71.1429	25.51764	3.93746
	CG	44	77.9318	17.05313	2.57086

**Table 2:** Independent Samples t test (groups’ pretests of LP, AI, and AE)

		Levene’s Test for Equality of Variances		t-test for Equality of Means				
		F	Sig.	t	df	Sig. (2-tailed)	Mean Difference	Std. Error Difference
LP Pre	Equal variances assumed	3.210	0.077	− 0.071	84	0.944	− 0.11580	1.63499
	Equal variances not assumed			− 0.071	81.589	0.943	− 0.11580	1.62663
AI Pre	Equal variances assumed	1.059	0.306	-1.527	84	0.130	-6.22186	4.07379
	Equal variances not assumed			-1.547	66.497	0.127	-6.22186	4.02112
AE Pre	Equal variances assumed	14.183	0.000	-1.457	84	0.149	-6.78896	4.66028
	Equal variances not assumed			-1.444	71.090	0.153	-6.78896	4.70243

Table 1 illustrates the mean scores of the two groups on the three pretests namely LP (Language Progress), AI, and AE. A close look at the mean scores shows that no remarkable difference can be seen between the two groups; however, to be more confidently ensured, the following table can be looked at:

Considering the Sig. (2-tailed) column in Table 2, all are higher than 0.05 (*p* > 0.05) revealing that there is no statistical difference/s between the two groups on the pretests. It can be concluded that the participants were at the same level of LP, AI, and AE before the treatment.

The third or last step was checking the performance of groups after receiving instruction. Thus, the MANOVA test was run.

The mean scores of both groups on the posttests of the three variables are illustrated in Table 3. It can be seen that the difference between the two groups on each variable is remarkable. Additional details on the results of multivariate tests are provided in Table 4:

**Table 3:** Descriptive statistics from posttests of LP, AI, and AE

	Groups	Mean	Std. Deviation	N
LP Posttest	EG	40.6190	16.49087	42
	CG	30.8182	7.49658	44
	Total	35.6047	13.56056	86
AI Posttest	EG	187.8571	23.37246	42
	CG	108.5227	45.93144	44
	Total	147.2674	54.05453	86
AE Posttest	EG	86.2143	10.75795	42
	CG	68.2955	17.85286	44
	Total	77.0465	17.26943	86

**Table 4:** Outcomes of multivariate tests

Effect		Value	F	Hypothesis df	Error df	Sig.	Partial Eta Squared
Intercept	Pillai’s Trace	0.224	7.620	3.000	79.000	0.000	0.224
	Wilks’ Lambda	0.776	7.620	3.000	79.000	0.000	0.224
	Hotelling’s Trace	0.289	7.620	3.000	79.000	0.000	0.224
	Roy’s Largest Root	0.289	7.620	3.000	79.000	0.000	0.224
Groups	Pillai’s Trace	0.684	56.883	3.000	79.000	0.000	0.684
	Wilks’ Lambda	0.316	56.883	3.000	79.000	0.000	0.684
	Hotelling’s Trace	2.160	56.883	3.000	79.000	0.000	0.684
	Roy’s Largest Root	2.160	56.883	3.000	79.000	0.000	0.684

The Wilk’s Lambda Test was employed to assess whether a significant difference existed between the two groups in the posttest, using a predetermined p-value of 0.05. The examination of Table 4 revealed that LP, AI, and AE all exhibited significant p values. The Partial Eta Squared value for each dependent variable was utilized to gauge the proportion of variance in the dependent variable attributable to the intended independent variable. All variables demonstrated values significant enough to suggest a notable difference between the two groups. To identify which of the three dependent variables contributed to the difference between the two groups, it is necessary to refer to Table 5:

**Table 5:** Test of between-subjects effects for LP, AI, AE

Source	Dependent Variable	Type III Sum of Squares	df	Mean Square	F	Sig.	Partial Eta Squared
Groups	LP Post	2088.505	1	2088.505	15.009	0.000	0.156
	AI Post	136366.273	1	136366.273	102.723	0.000	0.559
	AE Post	8487.761	1	8487.761	55.137	0.000	0.405

Given the multiple separate analyses conducted (Table 5), it is recommended to adopt a more stringent significance level to mitigate the risk of Type I error. A commonly employed method is the Bonferroni adjustment, which involves dividing the initial significance level (i.e., 0.05) by the number of analyses. In this instance, as there were three dependent variables, the significance level should be divided by three, resulting in a revised significance level of 0.016. Examining Table 5, under the Sig. column, the p-value for the three variables (0.000) was found to be less than 0.016. Consequently, it can be inferred that all three variables exhibited significant differences between the EG and CG due to the treatment administered to the EG learners.

## Discussion

The objective of this research was to investigate how the TS influences language progress, enjoyment, and immunity in virtual education. In order to achieve this goal, a quasi-experimental study was carried out with EFL students who were enrolled in English-language institutions and were taking online lessons in order to enhance their English Language abilities. EFL students who participated in online education with TS witnessed growth in their language acquisition. It was also discovered that TS promoted the development of enjoyment and productive immunity among the learners. Following is a discussion that provides an expanded description of the findings of the research.

The data suggest that teacher help and supervision may considerably improve student language learning. Attachment theory is a crucial aspect of developmental psychology since it explains the patterns of relationships between human beings [32]. This theory operates on the assumption that an individual’s interactions with others shape their behavior, which has the capacity to eventually become self-directed. The concept of emotional attachments among individuals has recently been included in the area of language education [32, 33]. This is done to highlight the importance of these attachments in fostering instructional connections, procedures, circumstances, and involvement in activities [33, 34]. EFL students build emotional relationships with their instructors and peers, which may be classified as comfortable or untrustworthy based on their degree of emotional sensitivity. As a result, the students do not feel compelled to suppress their ideas before expressing them. EFL students’ feeling of involvement helps them grow personally and academically, as well as improves their performance in online education.

In addition, the findings demonstrated that TS fosters productive immunity among students learning English via virtual instruction. In simpler words, the data revealed that TS exhibited positive and significant contributions to various aspects, including self-efficacy, resilience, attitude toward learning, coping skills, adaptability to change, and classroom effectiveness. These dimensions represent the sub-scales of learner immunity. Apart from that, the findings revealed that the modulation of the emotions of EFL learners while they attended online instruction reduced the likelihood of burnout occurring. It is possible to suggest, in conjunction with [19], that psychological well-being may be a contributing factor in the development of a productive configuration of immunity among the learners. Similarly, [15, 16, 35] contended that the emotional well-being of English learners is crucial for ensuring the cultivation of effective resilience and productive immunity.

A possible rationale for the results might be attributed to the notion that TS in virtual learning may contribute to a sense of equilibrium in the academic life of students. This, in turn, could result in increased excitement and involvement in teaching processes. Productive immunity, as described in self-organization theory, refers to a protective mechanism that helps individuals cope with various challenges encountered in their professional lives [14, 15]. The argument may be made that the tactics used in higher-order thinking skills promote self-awareness and self-organization, resulting in enhanced productivity and resilience. Productive immunity is fostered by emotional equilibrium, which is achieved via self-evaluation and self-organization [17].

Educators unquestionably occupy a pivotal position in language instruction programs as they furnish students with indispensable assistance that can foster their perseverance, readiness to express themselves in the second language, enthusiasm, involvement, favorable emotions, and overall experiences of learning [36–38]. TS allows students to track their learning progress and alter their efforts by receiving comments from teachers [39]. More crucially, the evaluative nature of instructors’ comments and recommendations on students’ present and desired levels of knowledge and performance contributes to improving learners’ learning processes and outcomes. EFL instructors may provide learners assessment help by providing them with constructive comments on their flaws, strengths, performance, and development. Additionally, they can engage in discussions with learners on aspects that contribute to their effectiveness or otherwise in learning English.

TS may be provided to students by EFL teachers in a variety of ways, including by presenting additional resources and materials that provide excellent instances and clear explanations, as well as by making helpful recommendations for improving their English. This form of assistance is regarded as an effective scaffolding mechanism, enabling instructors to provide guidance to learners in order to enhance their language learning process by using more effective tactics [40, 41]. As a result, experiencing a sense of achievement enhances their pleasure in academics and boosts their self-assurance. The results demonstrated that perceived support from educators has a significant positive impact on the fulfillment of fundamental psychological needs satisfaction, learning drive, and AE. Thus far, there have been limited studies undertaken to examine the connections between TS in virtual instruction and AE. The current study is among the first endeavors to do so.

Learners need instructors to provide them with multiple forms of assistance, encouragement, and helpful direction throughout the learning process. Students will exhibit positive learning habits and do well academically when they sense their instructors’ self-care and affection as well as their advice or assistance with their academic or personal issues. Numerous studies have shown that children who feel supported by their teachers are more likely to be self-assured, engaged, and persistent learners [42]. research demonstrated that students who were emotionally supported by their professors were more likely to be motivated to grasp the material, like learning, succeed academically, and display task-related behaviors. When it comes to their academic performance, students who believe their professors support them do better than those who do not [43, 44].

## Conclusion

All in all, the study’s conclusions lead to the proposal of some educational implications. Integrating psychological and mental aspects into the curriculum may enhance student access to course knowledge at any time and from any place, even outside of class. This is particularly important due to the rapid expansion of technology advancements and the increasing need for online and virtual educational settings. It is important to cultivate the positive psychology of students throughout their educational journey, as mentioned before. Both instructors and students need to understand the significance of TS, self-help principles, productive immunity, and the related implications for their mental and physical health. As a result, while establishing teaching techniques to improve students’ academic performance and accomplishment in comparable settings, language teachers should consider behavioral, emotional, and cognitive involvement. To adequately address it, instructional designers should provide specifically tailored training programs to particularly targeted training programs to assist professors in successfully improving the efficacy beliefs and engagement of students sustaining with the shift to emergency online learning.

In a way that is analogous to that of any experiential investigation, consideration needs to be given to the results of the current research while imposing some limitations. In the present investigation, a quasi-experimental approach was used, and the intact groups took part in the sampling procedures. In order to enhance the findings of the present inquiry, it is suggested that future research should make use of a variety of methodologies. Additionally, the average number of participants in both the EG and CG groups was quite low, which may have an impact on the extent to which the findings may be generalized. In the future, it will be necessary to conduct more research with additional students. Additionally, the potential impacts of the demographic information of the participants and the social-cultural variation among them were not investigated in this study. Future research may address these factors and investigate whether or not these variables influence the connections between TS, language achievement, AI, and AE in online instruction. This research was additionally constrained by its selected context (EFL instruction) and mode of teaching (virtual instruction). This study may be repeated by aspiring researchers in the future, but it is not necessarily restricted to online education.

### Electronic supplementary material

Below is the link to the electronic supplementary material.

Supplementary Material 1: Oxford Quick Placement Test.

## Data Availability

The dataset of the present study is available upon request from the corresponding author.

## Abbreviations

EFL: English as a foreign language
MANOVA: Multivariate Analysis of Variance
EG: Experimental Group
CG: Control Group
TS: Teacher Support
AE: Academic Enjoyment
AI: Academic Immunity
LP: Language Progress
OQPT: Oxford Quick Placement Test
AII: Academic Immunity Instrument
FLES: Foreign Language Enjoyment Scale

## Declarations

Written informed consent to participate in this study was provided by the participants and their legal parents. All the experiments in our study were conducted in accordance to the relevant guidelines and regulations of 1963 Helsinki declaration and its later amendments.
