# Supplementary material for: Teacher support in language learning: a picture of the effects on language progress, academic immunity, and academic enjoyment
Source: BMC Psychol. 2024 Mar 4;12:124. doi: 10.1186/s40359-024-01602-2 (PMC10913244; doi:10.1186/s40359-024-01602-2)
Supplement: Supplementary file 1 — Supplementary Material 1: Oxford Quick Placement Test. [file 40359_2024_1602_MOESM1_ESM.docx]

**Oxford Quick Placement Test**

Oxford University Press and University of Cambridge Local Examinations Syndicate

Name: ………………………………………………………………………….........

Date: …………………………………………………………………………...........

**Version 1**

**This test is divided into two parts:**

**Part One (Questions 1 – 40)**

**Part Two (Questions 41 – 60)**

**Do not start this part unless told to do so by your test supervisor.**

**Time: 30 minutes**

**Part 1**

**Questions 1 – 5**

Where can you see these notices?

For questions 1 to 5, mark one letter A, B or C on your Answer Sheet.

| **1.** | Please leave your room key at Reception | | A. in a shop  B. in a hotel  C. in a taxi |
| --- | --- | --- | --- |
| **2.** | Foreign money  changed here | | A. in a library  B. in a bank  C. in a police station |
| **3.** | AFTERNOON SHOW  BEGINS AT 2PM | | A. outside a theatre  B. outside a supermarket  C. outside a restaurant |
| **4.** | CLOSED FOR HOLIDAYS  Lessons start again on  the 8th January | | A. at a travel agent’s  B. at a music school  C. at a restaurant |
| **5.** | A. at a cinema  B. in a hotel  C. on a camp-site |  |  |

**Questions 6 – 10**

• In this section you must choose the word which best fits each space in the text below.

• For questions **6** to **10**, mark **one** letter **A**, **B** or **C** on your Answer Sheet.

**Scotland**

Scotland is the north part of the island of Great Britain. The Atlantic Ocean is on the west and the North Sea on the east. Some people **(6)** .................. Scotland speak a different language called Gaelic.

There are **(7)** .................. five million people in Scotland, and Edinburgh is **(8)** .................. most famous city.

Scotland has many mountains; the highest one is called ‘Ben Nevis’. In the south of Scotland, there are a lot of sheep. A long time ago, there **(9)** .................. many forests, but now there are only a **(10)** ................... . Scotland is only a small country, but it is quite beautiful.

**6. A.** on  **B.** in  **C.** at

**7. A.** about  **B.** between  **C.** among

**8. A.** his  **B.** your  **C.** its

**9. A.** is  **B.** were  **C.** was

**10. A.** few  **B.** little  **C.** lot

**Questions 11 – 20**

• In this section you must choose the word which best fits each space in the texts.

• For questions **11** to **20**, mark **one** letter **A**, **B**, **C** or **D** on your Answer Sheet.

**Alice Guy Blaché**

Alice Guy Blaché was the first female film director. She first became involved in cinema whilst working for the Gaumont Film Company in the late 1890s. This was a period of great change in the cinema and Alice was the first to use many new inventions, **(11)** .................. sound and color.

In 1907 Alice **(12)** ................... to New York where she started her own film company. She was **(13)** .................. successful, but, when Hollywood became the centre of the film world, the best days of the independent New York film companies were **(14)** ................... . When Alice died in 1968, hardly anybody **(15)** .................. her name.

**11. A.** bringing  **B.** including  **C.** containing  **D.** supporting

**12. A.** moved  **B.** ran  **C.** entered  **D.** transported

**13. A.** next  **B.** once  **C.** immediately **D.** recently

**14. A.** after **B.** down  **C.** behind  **D.** over

**15. A.** remembered  **B.** realized  **C.** reminded  **D.** repeated

**UFOs – do they exist?**

UFO is short for ‘unidentified flying object’. UFOs are popularly known as flying saucers, **(16)** .................that is often the **(17)** .................they are reported to be. The **(18)** .................. "flying saucers" were seen in 1947 by an American pilot, but experts who studied his claim decided it had been a trick of the light.

Even people experienced at watching the sky, **(19)** .................as pilots, report seeing UFOs. In 1978 a pilot reported a collection of UFOs off the coast of New Zealand. A television  **(20)** .................went up with the pilot and filmed the UFOs. Scientists studying this phenomenon later discovered that in this case they were simply lights on boats out fishing.

| **16.** | **A.** because | **B.** therefore | **C.** although | **D.** so |
| --- | --- | --- | --- | --- |
| **17.** | **A.** look | **B.** shape | **C.** size | **D.** type |
| **18.** | **A.** last | **B.** next | **C.** first | **D.** oldest |
| **19.** | **A**. like | **B.** that | **C.** so | **D.** such |
| **20.** | **A.** cameraman | **B.** director | **C.** actor | **D**. announcer |

**Questions 21 – 40**

• In this section you must choose the word or phrase which best completes each sentence.

• For questions **21** to **40**, mark **one** letter **A**, **B**, **C** or **D** on your Answer Sheet.

**21.** The teacher encouraged her students ....................to an English pen-friend.

**A.** should write **C.** wrote

**B.** write  **D.** to write

**22.** They spent a lot of time ....................at the pictures in the museum.

**A.** looking  **C.** to look

**B.** for looking **D.** to looking

**23.** Shirley enjoys science lessons, but all her experiments seem to ....................wrong.

**A.** turn  **C.** end

**B.** come  **D.** go

**24.** ....................from Michael, all the group arrived on time.

**A.** Except  **C.** Besides

**B.** Other  **D.** Apart

**25.**She ....................her neighbor’s children for the broken window.

**A.** accused  **C.** blamed

**B.** complained  **D.** denied

**26.** As I had missed the history lesson, my friend went .................the homework with me.

**A.** by  **C.** over

**B.** after  **D.** on

**27.** Whether she’s a good actress or not is a .................... of opinion.

**A.** matter **C.** point

**B.** subject  **D.** case

**28.** The decorated roof of the ancient palace was ....................up by four thin columns.

**A.** built  **C.** held

**B.** carried  **D.** supported

**29.** Would it ....................you if we came on Thursday?

**A.** agree  **C.** like

**B.** suit  **D.** fit

**30.** This form ....................be handed in until the end of the week.

**A.** doesn’t need  **C.** needn’t

**B.** doesn’t have **D.** hasn’t got

**31.** If you make a mistake when you are writing, just .................. it out with your pen.

**A.** cross  **C.** do

**B.** clear **D.** wipe

**32.** Although our opinions on many things .................... , we’re good friends.

**A.** differ  **C.** disagree

**B.** oppose  **D.** divide

**33.** This product must be eaten ....................two days of purchase.

**A.** by  **C.** within

**B.** before  **D.** under

**34.** The newspaper report contained ....................important information.

**A.** many  **C.** an

**B.** another  **D.** a lot of

**35.** Have you considered ....................to London?

**A.** move  **C.** to be moving

**B.** to move  **D.** moving

**36.** It can be a good idea for people who lead an active life to increase their ....................of vitamins.

**A.** upturn **C.** upkeep

**B.** input  **D.** intake

**37.** I thought there was a ..................... of jealousy in his reaction to my good fortune.

**A.** piece  **C.** shadow

**B.** part  **D.** touch

**38.** Why didn’t you ..................... that you were feeling ill?

**A.** advise **C.** remark

**B.** mention  **D.** tell

**39.** James was not sure exactly where his best interests ..................... .

**A.** stood **C.** lay

**B.** rested  **D.** centered

**40.** He’s still getting ....................the shock of losing his job.

**A.** across  **C.** over

**B.** by **D.** through

**Part 2**

Do not start this part unless told to do so by your test supervisor.

Questions 41 – 50

• In this section you must choose the word or phrase which best fits each space in the texts.

• For questions 41 to 50, mark one letter A, B, C or D on your Answer Sheet.

**The tallest buildings – SKYSCRAPERS**

Nowadays, skyscrapers can be found in most major cities of the world. A building which was many **(41)** ……………….. high was first called a skyscraper in the United States at the end of the 19^th^ century, and New York has perhaps the **(42)** …………….. skyscraper of them all, the Empire State Building. The **(43)** ……………….. beneath the streets of New York is rock, **(44)** ……………….. enough to take the heaviest load without sinking, and is therefore well-suited to bearing the **(45)** ……………….. of tall buildings.

**41. A.** stages  **B.** steps  **C.** stories  **D.** levels

**42. A.** first-rate  **B.** top-class **C.** well-built **D.** best-known

**43. A.** dirt  **B.** field  **C.** ground **D.** soil

**44. A.** hard  **B.** stiff  **C.** forceful  **D.** powerful

**45. A.** weight **B.** height **C.** size  **D.** scale

**SCRABBLE**

Scrabble is the world’s most popular word game. For its origins, we have to go back to the 1930s in the USA, when Alfred Butts, an architect, found himself out of **(46)** ……………….. . He decided that there was a **(47)** ………………. for a board game based on words and **(48)** ………………. to design one. Eventually he made a **(49)** ………………. from it, in spite of the fact that his original **(50)** ………………. was only three cents a game.

| **46.** | **A.** earning | **B.** work | **C.** income | **D.** job |
| --- | --- | --- | --- | --- |
| **47.** | **A.** market | **B.** purchase | **C.** commerce | **D.** sale |
| **48.** | **A.** took up | **B.** set out | **C.** made for | **D.** got round |
| **49.** | **A.** wealth | **B.** fund | **C.** cash | **D.** fortune |
| **50.** | **A.** receipt | **B.** benefit | **C.** profit | **D.** allowance |

**Questions 51 – 60**

• In this section you must choose the word or phrase which best completes each sentence.

• For questions **51** to **60**, mark **one** letter **A**, **B**, **C** or **D** on your Answer Sheet.

**51.** Roger’s manager ................... to make him stay late if he hadn’t finished the work.

**A.** insisted  **C.** threatened

**B.** warned  **D.** announced

**52.** By the time he has finished his week’s work, John has hardly ................... energy left for the weekend.

**A.** any  **C.** no

**B.** much **D.** same

**53.** As the game ....................to a close, disappointed spectators started to leave.

**A.** led  **C.** approached

**B.** neared  **D.** drew

**54.** I don’t remember ....................the front door when I left home this morning.

**A.** to lock  **C.** locked

**B.** locking  **D.** to have locked

**55.** I ..............to other people borrowing my books: they always forget to return them.

**A.** disagree **C.** dislike

**B.** avoid  **D.** object

**56.** Andrew’s attempts to get into the swimming team have not ....................with much success.

**A.** associated  **C.** joined

**B.** concluded  **D.** met

**57.** Although Harry had obviously read the newspaper article carefully, he didn’t seem to have.................... the main point.

**A.** grasped  **C.** clasped

**B.** clutched  **D.** gripped

**58.** A lot of the views put forward in the documentary were open to .................... .

**A.** enquiry **C.** question

**B.** query  **D.** wonder

**59.** The new college ....................for the needs of students with a variety of learning backgrounds.

**A.** deals  **C.** furnishes

**B.** supplies **D.** caters

**60.** I find the times of English meals very strange – I’m not used ................ dinner at 6pm.

**A.** to have  **C.** having

**B.** to having  **D.** have
